# A position anchor sinks the double-drift illusion

**DOI:** 10.1167/jov.21.6.3

**Published:** 2021-06-09

**Authors:** Sharif Saleki, Patrick Cavanagh, Peter U. Tse

**Affiliations:** 1 Department of Psychological and Brain Sciences, Dartmouth College, Hanover, NH, USA; 2 Centre for Vision Research, York University, Toronto, Ontario, Canada; 3 Department of Psychology, Glendon College, Toronto, Ontario, Canada

**Keywords:** positional uncertainty, visual motion perception, double-drift illusion, motion-induced position shift

## Abstract

When the internal texture of a Gabor patch moves orthogonally to its envelope's motion, the perceived path, viewed in the periphery, shifts dramatically in position, and direction relative to the true path (the double-drift illusion). Here, we examine positional uncertainty as a critical factor underlying this illusory shift. We presented participants with an anchoring line at different distances from the drifting Gabor's physical path. Our results indicate that placing an anchor (a fixed line) close to the Gabor's path halved the magnitude of the illusion. This suppression was symmetrical for anchors placed on either side of the Gabor. In a second experiment, we used crowding to degrade the anchoring line's position information by embedding it in a set of parallel lines. In this case, despite the presence of the same lines that reduced the illusion when presented in isolation, the illusory shift was now largely restored. We suggest that the adjacent lines crowded each other, reducing their positional certainty, and thus their ability to anchor the location of the moving Gabor. These findings indicate that the positional uncertainty of the equiluminant Gabor patch is critical for the illusory position offset.

## Introduction

In the case of visual illusions of position, the conscious perception of an object's position differs from the position indicated by the receptive fields in early visual cortex that respond to the object. Motion-Induced Position Shifts (MIPS) occur when motion signals influence a target's perceived location. For instance, flashing a stimulus briefly at the time of reversal of an oscillatory motion changes the perceived position of the flash, shifting it in the direction of subsequent motion (flash grab; Cavanagh & Anstis, 2013). Such illusions demonstrate that position is not determined solely by the locations of the receptive fields activated in early visual areas. Motion extrapolation mechanisms that compensate for neural processing delays have been suggested as the source of these misperceptions (Hogendoorn, 2020; Nijhawan, 1994; van Heusden, Rolds, Cavanagh, & Hogendoorn, 2018).

The double-drift illusion (Lisi & Cavanagh, 2015), also known as the infinite regress (Tse & Hsieh, 2006) or the curveball illusion (Shapiro, Lu, Huang, Knight, & Ennis, 2010), presents a different and unique case of illusory perception of position. In this illusion, a Gabor patch moves in one direction while its internal texture moves in the direction orthogonal to its path. This results in a dramatic shift in perceived position and direction. The illusion appears to emerge at a high level in the visual processing hierarchy as the early visual cortex shows no functional magnetic resonance imaging (fMRI) activation corresponding to the perceived path of the double-drift stimulus (Liu, Yu, Tse, & Cavanagh, 2019). In contrast, the influence of other varieties of MIPS, such as the flash grab, can already be seen in V1 (Ge, Zhou, Qian, Zhang, Wang, & He, 2020; Kohler, Cavanagh, & Tse, 2017). Additionally, unlike the small position shifts of a stationary Gabor patch that has only internal texture motion (De Valois & De Valois, 1991), once the Gabor itself is also set in motion in the double drift stimulus, the magnitude of the illusion increases dramatically; its offset in direction from the veridical direction can be more than 45 degrees (Liu, Tse, & Cavanagh, 2018). This increase in effect is due to accumulation of position shifts over a much longer period of time than that is seen for the stationary Gabor (Arnold, Thompson, & Johnston, 2007; Chung, Patel, Bedell, & Yilmaz, 2007). This accumulation only takes place when the background is equiluminant with the mean luminance of the Gabor patch, presumably because position signals are weak in the absence of luminance contrast. Under these circumstances, the perceived trajectory of the Gabor patch follows a direction consistent with a vector combination of its internal and external motions (Cavanagh & Tse, 2019; Tse & Hsieh, 2006).

The double-drift illusion likely arises because of the positional uncertainty of the Gabor patch (Gurnsey & Biard, 2012; Kwon, Tadin, & Knill, 2015). The illusion is lost when the mean luminance of the drifting Gabor differs from the background (Cavanagh & Tse, 2019; Gurnsey & Biard, 2012). Computational object-tracking models that aim to predict the illusory shift of the illusion (Kwon et al., 2015) also assume that the visual system relies more on motion signals in situations where position is noisy and unreliable. We explored this uncertainty principle by manipulating the degree of positional certainty. We did this by placing an anchoring line at various distances from the drifting patch in order to test the hypothesis that reducing positional uncertainty leads to lower illusory shifts. In a second experiment, we degraded the positional certainty of the same anchoring lines used in experiment 1 by crowding it with flanking lines. In this case, the same line, when embedded within the grid of lines, may not be able to anchor the double-drift illusion because crowding has rendered its own position uncertain.

## Experiment I: Position anchoring

### Materials and methods

#### Participants

Seventeen Dartmouth college undergraduate students participated in this experiment. All participants were naïve to the purpose of the experiment and had normal or corrected-normal vision. Participants signed a written informed consent approved by the Committee for the Protection of Human Subjects at Dartmouth College and were compensated for their time with course credit.

#### Apparatus

The experiment was written in Python using the PsychoPy library for psychophysics (Peirce, 2007). Stimuli were displayed on a monitor with 1024 × 768 pixel resolution and 60 Hz refresh rate by a computer running Ubuntu 16.04 LTS. Viewing distance was fixed at 57 cm using a chin rest during the experiment and there were no other sources of lighting in the room except the display screen. The display had a mid-gray background and responses were collected with a mouse and keyboard.

#### Stimuli

**Figure 1.**
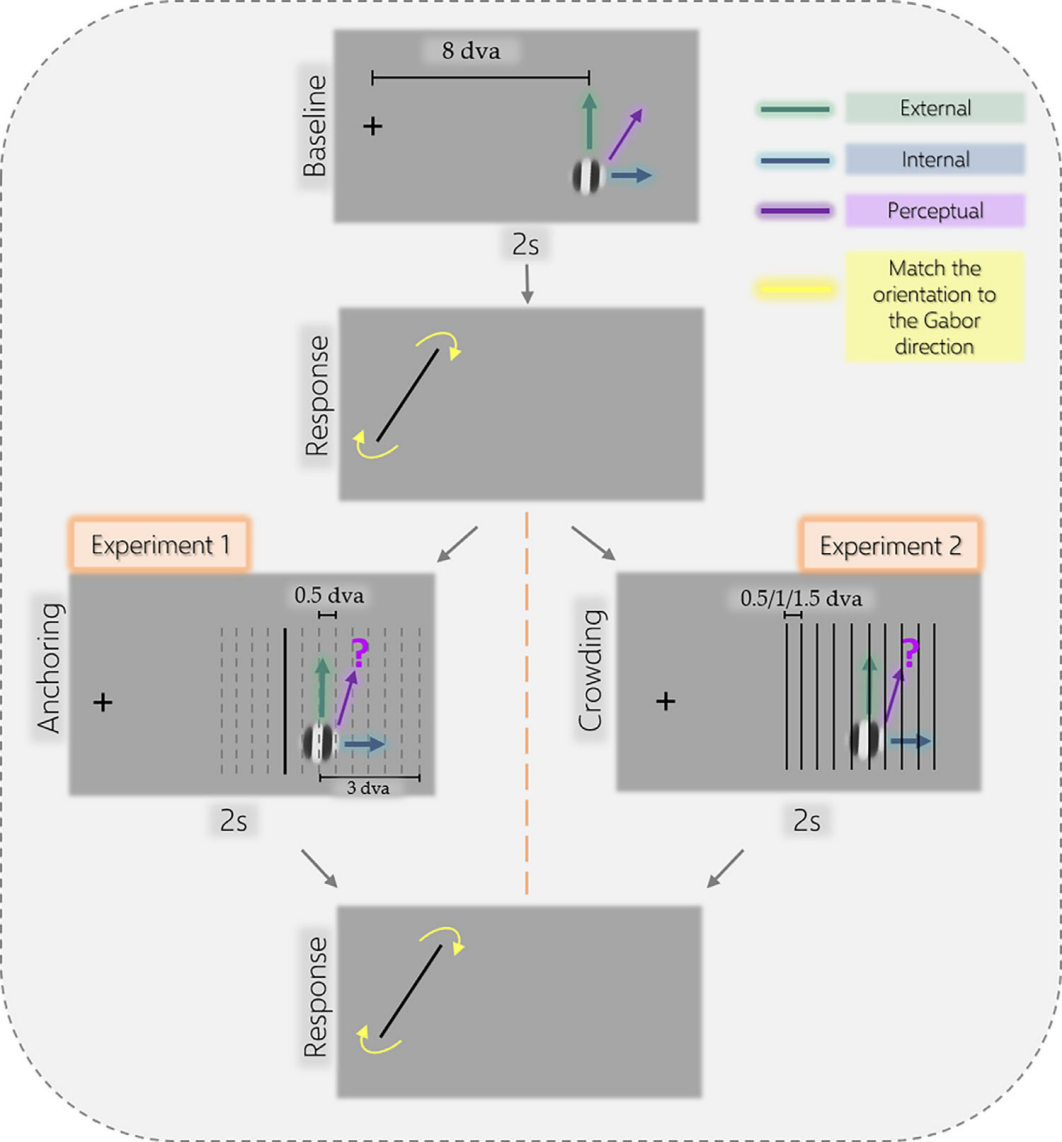
A traversing Gabor patch with texture and envelope component motions was shown on each trial. In the baseline condition no anchor was displayed to find the maximum illusory shift perceived by the participant. In experiment 1, a single vertical high-contrast line (at one of the 13 possible locations demonstrated by dashed lines; ranging from 3 dva from the left of the Gabor to 3 dva to its right, in multiples of 0.5) was shown alongside the drifting patch. On the experimental trials of experiment two, a collection of lines was displayed that varied in their distance from each other on each condition. After each trial, a response line appeared in place of the fixation cross. Participants adjusted the orientation of this line to match the perceived path of the viewed stimulus.

As shown in Figure 1, a fixation cross 0.3 degrees of visual angle (dva) in width was displayed on the screen, offset 5 dva left of the center of the screen along its horizontal axis. The stimulus was a sinusoidal grating within a Gaussian envelope 1 dva in diameter, with a spatial frequency of 1.2 cycle/dva, and 70% contrast. It was positioned 3 dva right of the center of the screen (8 dva eccentricity from fixation). On each trial, the Gabor patch traversed a linear 2.8-dva vertical path (parallel to the internal texture's orientation) at a speed of 4.2 dva/s, for 2 seconds (4 up and down transits of the path). The internal grating also had a rightward drift of 5.4 Hz during the upward movement of the Gabor patch, and a leftward drift at the same temporal frequency during the downward trajectory. Many different values for Gabor properties, eccentricity, and speed were examined in pilot experiments and the mentioned values were chosen to obtain a large and reliable illusion size. On the experimental trials, a black vertical line 0.2 dva in width (the anchor) and 2.8 dva long was also displayed at a fixed pseudo-random position.

#### Procedure

Participants were instructed to fixate the fixation cross on the display during each trial and avoid making head movements throughout the experiment. Each trial was self-initiated by pressing a key. In the baseline condition, the stimulus consisted solely of the drifting patch. The closest possible anchor in this condition was the edge of the display positioned at 17 dva to the right of the Gabor patch. On the experimental trials, an anchoring line appeared on either side of the Gabor parallel to its path. The separation of the anchoring line from the Gabor's path took one of 13 values from -3 dva to 3 dva (in steps of 0.5) where at 0 dva the anchoring line was on the Gabor's path. Following stimulus presentation (2 s), the fixation cross was removed and was replaced by a response bar. Participants changed the orientation of the response bar using the mouse wheel until its orientation matched the perceived path orientation of the double-drift illusion that they had just viewed. A keypress was used to indicate the end of reporting, at which point the next trial began. Each participant completed 210 trials in one session.

#### Analysis

One-sample *t*-tests were used to compare the reported perceived angle of the double-drift to its veridical trajectory (vertical, or 0 degrees deviation angle) for each condition. We used a one-way repeated measure ANOVA to compare the average magnitude of the reported illusion across conditions (1 baseline condition and 13 conditions for the different line positions). Post hoc *t*-tests revealed the distances at which anchors significantly interfered with the illusion. A false discovery rate (FDR) correction (Benjamini & Hochberg, 1995) was used to adjust *p* values for multiple *t*-tests.

Our stimulus always started with an upward external motion and rightward texture motion, resulting in a perceived trajectory toward the upper right corner of the display (see Figure 1). Therefore, anchoring lines that were placed to the right of the drifting patch would look like a barrier, whereas the ones on the other side would not induce the same perceptual impression. We used specific contrasts to examine the effect of placement of the anchors on left versus the right side of the Gabor stimulus.

## Results

In the baseline condition with no anchoring line, participants reported an illusory oblique path that deviated significantly from the veridical trajectory (0 degrees) by 28.09 ± 1.21 degrees on average (*t*(16) = 8.7, *p* < 0.001), indicating that the participants indeed perceived the illusion.

**Figure 2.**
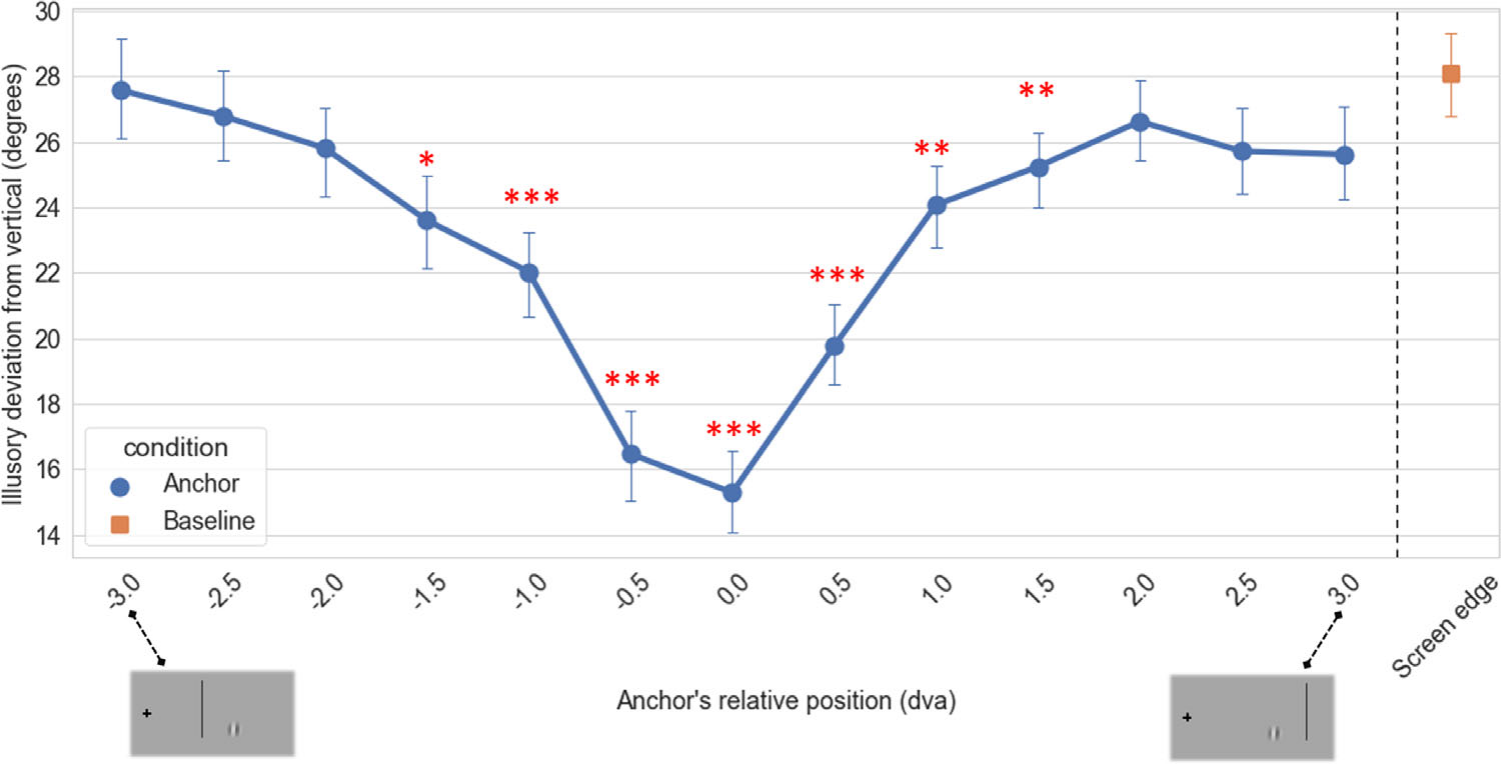
An anchoring line with closer than 1.5 dva distance to the double-drift's actual path (0 dva distance anchor lies along the Gabor's path) dramatically reduced the magnitude of the illusion compared to more distant placements. Error bars indicate standard error of the mean. Asterisks indicate significant difference from baseline (no flanking lines) condition.

A repeated-measures, one-way ANOVA with anchor position (including the baseline condition) as a within-subject factor showed that the anchors had a significant effect on the magnitude of the perceived illusion (*F*(13,208) = 10.25, Greenhouse-Geisser *p* < 0.001, ƞ^2^ = 0.39). Post hoc analysis showed that among the experimental conditions, anchors positioned less than 1.5 dva away from the double-drift path (1.0 and 0.5 dva to the left and right, as well as 0 dva, which corresponds to anchors placed on the Gabor's path) significantly reduced the magnitude of the illusion compared to the baseline condition (all FDR-corrected *p* values < 0.01; Figure 2). Additionally, the anchoring line that was placed 1.5 dva to the right of the Gabor (away from fixation) strongly reduced the perceived angle of the trajectory from baseline (*t*(16) = 3.55, FDR-corrected *p* < 0.01). The line placed at the other side of the Gabor at the same distance (1.5 dva to the left) also significantly reduced the magnitude of the illusion, but to a lesser extent (*t*(16) = 2.92, FDR-corrected *p* < 0.05). Even though corresponding lines on each side of the Gabor had different levels of significance, there was no effect of the anchor's side; a contrast for lines on the left versus the right side of the Gabor was not significant (*t*(16) = 0.75, *p* = 0.46).

## Experiment II: Crowding

### Materials and methods

#### Participants

Five Dartmouth college graduate students (different from those who participated in experiment 1) participated in the second experiment. Participants signed an informed consent approved by the Committee for the Protection of Human Subjects at Dartmouth College and reported to have normal or corrected-normal vision.

#### Apparatus

The same setup was used for this experiment as experiment one.

#### Stimuli

The fixation cross and the double-drift stimulus were the same as in the previous experiment. On the experimental trials, however, instead of one solid black line, the Gabor patch was overlayed with a grid of 10, 15, or 30 lines (see Figure 1). Each line had the same width, length, and orientation as in the previous experiment and all lines in each grid were spaced equally. The grid was centered on the Gabor patch and the distance between the lines for each trial was pseudo-randomly selected from 0.5, 1, and 1.5 dva (for 30, 15, and 10 lines) so that the whole grid spanned 14.5, 14, and 13.5 dva on the horizontal axis, respectively. These overall widths were chosen to be roughly the same across conditions while keeping the outlines of the grid (left and right edges) well outside of the interference region found in experiment one, so the only sources of any anchoring effect would be the lines near the Gabor patch.

#### Procedure

Data were collected using the same procedure as experiment one. Each participant completed 200 trials in this experiment.

#### Analysis

Baseline deviations from the veridical path were tested using the same method as the previous experiment (1-sample *t*-tests with FDR correction for multiple comparison). A repeated measure ANOVA was performed to find the effect of the grid on the magnitude of the illusion.

## Results

In each grid (30, 15, or 10 lines), the lines closest to the Gabor's path corresponded to lines positioned at distances that significantly reduced the illusion magnitude in experiment one (0.0, 0.5, 1, or 1.5 dva). The relatively large effect size observed in that experiment (η^2^ = 0.39) indicated the strong influence of these anchoring lines on the illusion size. However, the presence of the grid restored the illusion size to a level comparable to no line being present. A one-way repeated measure ANOVA analysis here did not show a significant effect of the presence of the grid, at any of its spacings (*F*(3, 12) = 1.99, Greenhouse-Geisser-corrected *p* = 0.22).

## Discussion

**Figure 3.**
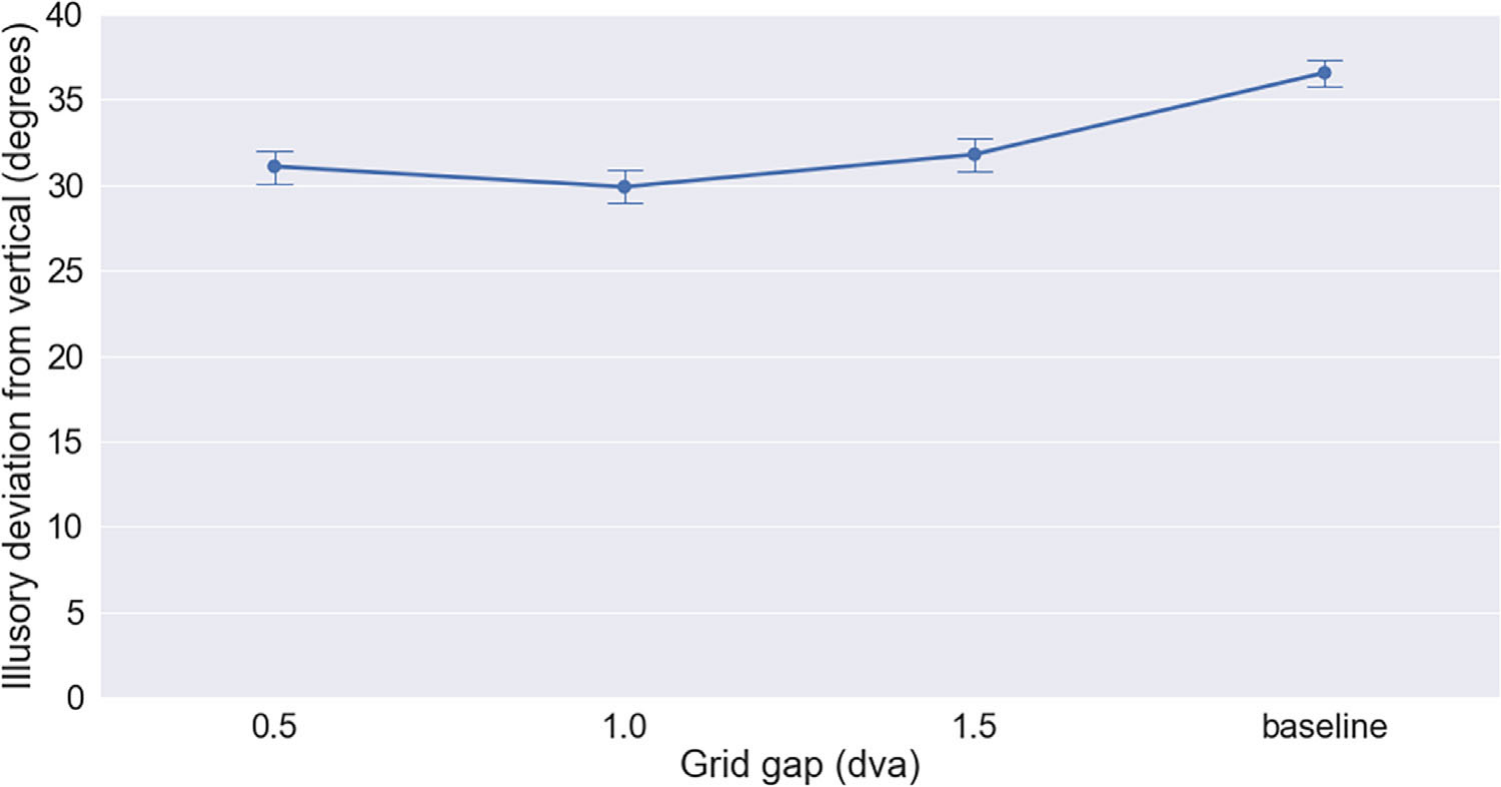
Multiple anchor lines displayed together act as a surface, texture, or grid. The grid's effect on the illusory deviation is much less than that of a single anchor although the same anchoring line was present within the grid. Distance between grid lines was selected to match the distances of anchors that had the highest effect on the illusion. None of the grid conditions resulted in a significant reduction of the illusory angle.

Here, we show that a nearby static line reduces the illusory shift of the double-drift Gabor away from its veridical path. The magnitude of this effect changes as a function of distance, such that closer anchors decrease the magnitude of the illusion to a greater extent—down to a 46% reduction in the case that an overlapping line reduced the illusory shift from the 28 degrees (baseline) to 16 degrees. In addition, it appears that anchoring is driven by the absolute value of the distance between the object and the Gabor, and the effect is symmetrical, being equally strong whether the Gabor appears to be approaching the anchor or moving away from it. Results from our second experiment revealed that introducing additional parallel flanking lines do not elicit more anchoring effect, as might be expected, that adding more stationary lines would provide more positional certainty. Adding multiple lines instead brings back the illusory shift (Figure 3).

Although we measured the perceived direction of the Gabor patch in our experiments, this illusion is an effect on location (Lisi & Cavanagh, 2015). It has been demonstrated that increasing the contrast between the double-drift and its background removes the illusory shift (Cavanagh & Tse, 2019; Gurnsey & Biard, 2012), suggesting that this illusion relies on positional uncertainty of the Gabor patch (there is no illusion when the position information is reliable enough to determine where the Gabor is seen and where it is going). Our study also supports this positional uncertainty principle; position signals play a central role in driving the double-drift illusion, such that in the presence of unambiguous positional information, the visual system becomes less reliant on the object's motion (a vector combination of envelope and texture component motions; Cavanagh & Tse, 2019) to estimate its position. Stationary anchors that are near the double-drift stimulus increase the reliability of position information of objects in that region of space, reducing the magnitude of the illusion. In contrast, stationary lines that are far from the Gabor's path do not impose the same effect on the illusion (farther than 1.5 dva). It is important to note that adding a line does not change the vector combination that underlies the direction of the illusory motion—its direction is independent of the presence or absence of the line. However, the line increases the certainty of the incoming position signal and thereby influences the trade-off between this signal and the position predicted by the combined motion vector.

Our results show that anchors that are more than a few degrees from the Gabor's path have little or no effect on the illusion (for a Gabor with 1 dva diameter). Furthermore, the overall placement of the Gabor to the left or the right side of the illusory path of the drifting Gabor had no effect, as shown by the symmetry in Figure 2^3^. Rather, it is the absolute distance that acts to reduce the magnitude of the illusion. Our stimulus started from the bottom point of its path always with rightward internal drift and upward external motion, resulting in an oblique path to the right, away from the physical path. Both the internal and external motion directions then reversed when the Gabor reached the end point of its path at the top, tracing the same illusory oblique path back to its starting position. This position shift to the right side of the physical path could make it more likely for the Gabor to run into the anchoring line if the line were placed on the right of the stimulus. However, the mentioned symmetry of the observed anchoring effect does not support this perceptual “blocking” of the illusory path. Because the stimulus traversed its path four times, this symmetry might be due to centering of the illusory path around the midpoint of the physical path and extending equally on both sides after the first passing of the path. Alternatively, if the illusory path did remain on the right side of the physical path throughout each trial, we could infer that interference occurs between the anchoring line and the physical path, not the perceived path.

Our second experiment further strengthens the importance of the positional uncertainty argument. In this experiment, we used the same anchoring lines that significantly reduced the illusory shift, at the same distances from the Gabor that had the largest effect in experiment one in terms of reducing the magnitude of the double-drift illusion. However, the presence of multiple parallel lines triggered a crowding effect (Bouma, 1970; Intriligator & Cavanagh, 2001) that reduced the positional certainty of all of the lines. The crowding effect arises when items are spaced at less than one third their eccentricity (Bouma, 1970). If the position information of each anchor was simply summed with its flanks, we should have observed a more dramatic decrease in the illusion. However, our results showed a substantial return of the illusory shift, which was no longer significantly different from the noncrowded baseline with no lines present.

Taken together, the first and second experiment of this study show that, in the presence of uncertain position information, a drifting grating patch follows an illusory trajectory that is driven strongly by its internal motion. Although the starting point of motion may be the physical position, drift in the direction of the internal motion accumulates over hundreds of milliseconds, leading to large misperceptions of position and direction. Our findings about the role of positional uncertainty are consistent with ideas proposed by Kwon et al. (2015) in their object tracking model. However, although our stimulus was displayed for 500 ms, its trajectory did not saturate or appear curved (see Movie 1) as predicted by that model, so we cannot make a direct comparison of our data to the model's predictions.

Our data do not speak to the question of how such accumulation of position errors occurs. This remains a core unsolved problem posed by the double-drift illusion. Nevertheless, we show that the origins of this illusion reside in visual processes that estimate position based on motion signals when position uncertainty is high.

## Supplementary Material

Supplement 1
